# The effect of an mGluR5 inhibitor on procedural memory and avoidance discrimination impairments in *Fmr1* KO mice

**DOI:** 10.1111/j.1601-183X.2011.00763.x

**Published:** 2012-04

**Authors:** M F Vinueza Veloz, R A M Buijsen, R Willemsen, A Cupido, L W J Bosman, S K E Koekkoek, J W Potters, B A Oostra, C I De Zeeuw

**Affiliations:** † Department of NeuroscienceRotterdam; ‡ Department of Clinical Genetics, Erasmus Medical CenterRotterdam; § Netherlands Institute for Neuroscience, Royal Academy of Arts and SciencesAmsterdam, The Netherlands

**Keywords:** Avoidance behavior, cue recognition, Erasmus Ladder, *Fmr1* KO, Fragile X syndrome, locomotion, mGluR5 inhibitor, motor learning, procedural memory formation

## Abstract

Fragile X syndrome (FXS) is the most common inherited form of intellectual disability. Patients with FXS do not only suffer from cognitive problems, but also from abnormalities/deficits in procedural memory formation. It has been proposed that a lack of fragile X mental retardation protein (FMRP) leads to altered long-term plasticity by deregulation of various translational processes at the synapses, and that part of these impairments might be rescued by the inhibition of type I metabotropic glutamate receptors (mGluRs). We recently developed the Erasmus Ladder, which allows us to test, without any invasive approaches, simultaneously, both procedural memory formation and avoidance behavior during unperturbed and perturbed locomotion in mice. Here, we investigated the impact of a potent and selective mGluR5 inhibitor (Fenobam) on the behavior of *Fmr1* KO mice during the Erasmus Ladder task. *Fmr1* KO mice showed deficits in associative motor learning as well as avoidance behavior, both of which were rescued by intraperitoneal administration of Fenobam. While the *Fmr1* KO mice did benefit from the treatment, control littermates suffered from a significant negative side effect in that their motor learning skills, but not their avoidance behavior, were significantly affected. On the basis of these studies in the FXS animal model, it may be worthwhile to investigate the effects of mGluR inhibitors on both the cognitive functions and procedural skills in FXS patients. However, the use of mGluR inhibitors appears to be strongly contraindicated in healthy controls or non-FXS patients with intellectual disability.

Fragile X syndrome (FXS) is the most common genetic form of mental impairment (WHO 1996), affecting approximately 1 in 4000 males (Crawford *et al.* 2002; de Vries *et al.* 1997; Patsalis *et al.* 1999; Youings *et al.* 2000) and 1 in 6000 females worldwide (Crawford *et al.* 2001). In nearly all cases, the observed mutation is an expansion of a CGG trinucleotide repeat (>200) in the 5′-untranslated region (UTR) region of the fragile X mental retardation gene (*FMR1*) (Oberle *et al.* 1991; Verkerk *et al.* 1991). As a consequence, the *FMR1* gene is methylated and cannot be transcribed into mRNA, causing the absence of fragile X mental retardation protein (FMRP) (Oostra & Willemsen 2009). Besides physical characteristics such as macro-orchidism and facial features (Pfeiffer & Huber 2009), the symptoms of FXS include general deficits in cognitive processing (Van der Molen *et al.* 2010), abnormalities in procedural memory formation (Koekkoek *et al.* 2005), social anxiety and autistic-like behavior (Sabaratnam *et al.* 2003).

FMRP, which is an RNA binding protein (Schaeffer *et al.* 2003), is present in the postsynaptic compartment and locally synthesized upon mGluR activation (Weiler *et al.* 1997). As an RNA binding protein, FMRP is thought to repress the translation of target mRNAs that are important for receptor recycling in the postsynaptic dendritic spines (Levenga *et al.* 2010; Pfeiffer & Huber 2009). The absence of FMRP induces increased translation of a subset of mRNAs, which results in altered receptor trafficking dynamics. Internalization of *α*-amino-3-hydroxy-5-methyl-4-isoxazole-propionic acid (AMPA) receptors may facilitate long-term plasticity and is stimulated by the synthesis of novel proteins after the activation of mGluRs (Snyder *et al.* 2001). Accordingly, the ‘mGluR theory of FXS' suggests that the neurobiological and psychiatric symptoms of FXS result from an exaggerated AMPA receptor internalization triggered by mGluR activation (Bear *et al.* 2004). As a consequence the mGluR theory has directed research toward the use of mGluR antagonists to treat FXS.

A ladder rung task provides comprehensive assessment for skilled limb movements in mice (Farr *et al.* 2006; Hunsaker *et al.* 2011). As FXS patients suffer from both motor abnormalities and cognitive deficits (Koekkoek *et al.* 2005; Sabaratnam *et al.* 2003; Van der Molen *et al.* 2010), we subjected *Fmr1* KO mice to the Erasmus Ladder test, which allows a quantitative assay for both categories of symptoms. With regard to the motor abnormalities, the Erasmus Ladder test offers sensitive measurements for locomotion learning controlled by the olivocerebellar system (Renier *et al.* 2010; Van Der Giessen *et al.* 2008; Van der Vaart *et al.* 2011). For example, blockage of electrotonic coupling in the inferior olive results in impaired learning-dependent timing of locomotion steps during classical delay conditioning (Van Der Giessen *et al.* 2008). With regard to avoidance behavior, which is mainly controlled by limbic and basal ganglia systems (Ermisch *et al.* 1986; Ursin 1965), the Erasmus Ladder task can test the ability of mice to temporarily prevent their exposure to the stressful situation on the ladder that is created by unexpectedly lowering or rising one of the rungs; because of the presence of such an unconditioning stimulus (US), mice try to avoid the US by waiting inside the shelter box as long as possible and thus inhibit their reaction to the cues of departure (Ursin (1965)). Moreover, different from other tests such as eyeblink conditioning, in which *Fmr1* KO mice also show a phenotype (Chen & Toth 2001; Paylor *et al.* 2008; Spencer *et al.* 2006), the Erasmus Ladder test does not require any surgical intervention and allows drug screening at an automated, medium-throughput level. Thus, because of the technical advantages, we tried to test the ‘mGluR theory of FXS' by investigating the impact of a specific mGluR negative modulator, Fenobam, on the behavior of mice lacking FMRP (‘fragile X mental retardation 1 knockouts' or ‘*Fmr1* KO mice’) using the multifunctional, motor-cognitive assay on the Erasmus Ladder.

## Methods

### Animals

*Fmr1* KO mice were obtained by crossing FVB/Ant x het *Fmr1* KO(2) to test hybrid mice with 50% FVB/Ant and 50% C57Bl/6 contribution. Because the FVB/Ant strain is pigmented and devoid of the genetic predisposition to retinal degeneration of the FVB/N strain, these mice show clear visual evoked potential in the presence of normal eye histology and improved performance in the Morris water maze test (Errijgers *et al.* 2007). The *Fmr1* KO(2) line, unlike the first generation of *Fmr1* KO model, does not express any FMRP and lacks detectable *Fmr1* transcripts (Mientjes *et al.* 2006). Both lines were inbred (>10 times backcrossed). All mice were male between 12 and 26 weeks of age and were single housed. Mice were allowed to have free access to standard laboratory food and water. They were left on a 12 h light/dark cycle. As required by Dutch legislation, all experiments were approved in advance by the Institutional Animal Welfare Committee (Erasmus MC, Rotterdam, The Netherlands).

### Treatment

Fenobam [*N*-(3-chlorophenyl)-*N*’-(4,5-dihydro-1-methyl-4-oxo-1*H*-imi dazole-2-yl)urea], which is a clinically validated non-benzodiazepine anxiolytic drug, is a selective and potent mGluR5 receptor antagonist acting at an allosteric modulatory site shared with 2-methyl-6-(phenylethynyl)-pyridine (MPEP) (Porter *et al.* 2005). Similar to MPEP, Fenobam acts in a noncompetitive manner and shows inverse agonist properties, blocking 66% of mGluR5 receptor basal activity given at a dose of 10–30 mg/kg orally. Fenobam (Sigma-Aldrich, St. Louis, MO, USA) was injected intraperitoneally at a dose of 30 mg/kg 30 min before each associative motor learning session, using methyl cellulose (MC) as dissolvent. For the motor learning and avoidance discrimination tasks *Fmr1* KO and wild-type (WT) mice were assigned to different groups: one treated with Fenobam in MC and another one treated with the vehicle (MC only). In addition, we also tested the performance of *Fmr1* KO and WT littermates without any application as control.

### The Erasmus Ladder

The Erasmus Ladder is a fully automated test for detecting motor performance, associative motor learning deficits and cognitive phenotypes in mouse models. The Erasmus Ladder consists of a horizontal ladder in between two shelter boxes, which are equipped with a bright white LED spotlight in the roof and two pressurized air outlets (Pneumax, Gosport, UK). Both, light and air stimuli are used as cues for departure. In addition, one of the air outlets is used to control the speed of the mice and to prevent them from leaving the shelter box at the opposite side and crossing the ladder at unwanted moments (which we call ‘escape’). The ladder has 2 × 37 rungs for the left and right side. All rungs are equipped with pressure sensors (produced at Erasmus MC), which are continuously monitored and which can be used to register and analyze the walking pattern of the mouse instantaneously. Moreover, based upon the prediction of the walking pattern, the rungs can be moved up or down by a high-speed pneumatic slide (Pneumax) with a maximum of 13 mm at any moment in time. The computer system (National Instruments, Austin, TX, USA), which runs the real-time system recording sensor data, adjusts air pressure, predicts future touches, calculates interventions, repositions slides and stores data, operates in a fixed cycle of 2 ms. Details of the device and its operations have been published (Van Der Giessen *et al.* 2008).

During the first 4 days (‘unperturbed sessions'), mice were trained with the even-numbered rungs on the left side and the odd-numbered rungs on the right side in a descended position so as to create an alternated stepping pattern with 30-mm gaps. Mice were trained to walk the ladder for 72 runs per day. We calculated the number of missteps that were sensed by the descended rungs, and steptime, which is defined as the time needed to place one of the front paws from one rung to the other (i.e. onset of touch until onset of following touch). Associative motor learning trials (‘perturbed sessions') started on day 5 using a 15 kHz tone as conditioning stimulus (CS; gradually increasing over 20 ms to 100 dB and lasting up to 300 ms; Voltcraft, Barking, UK) and a rising rung as the US (ascending 12 mm). The interstimulus interval was fixed at 285 ms. To keep this time period constant, we observed in real-time the speed of the mouse and calculated which rung should rise. Mice typically learn that increasing walking speed avoids being hit by the rung, so mice will decrease their pre-steptime (nearest steptime before the onset of the US) and post-steptime (nearest steptime after the onset of the US; i.e. not the average of all steptimes after onset of US) through the sessions.

**Figure 1:**
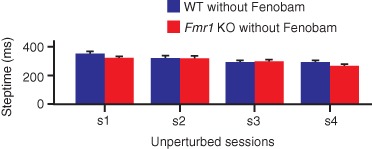
Motor performance of *Fmr1* KO mice Motor performance was tested with the use of the Erasmus Ladder by calculating the average steptimes for every unperturbed session (s1–s4). *Fmr1* KO (*n* = 35) and their WT (*n* = 41) littermates did not show significant differences in steptimes (*P* = 0.810; repeated measures anova). Error bars represent standard error of the mean.

**Figure 2:**
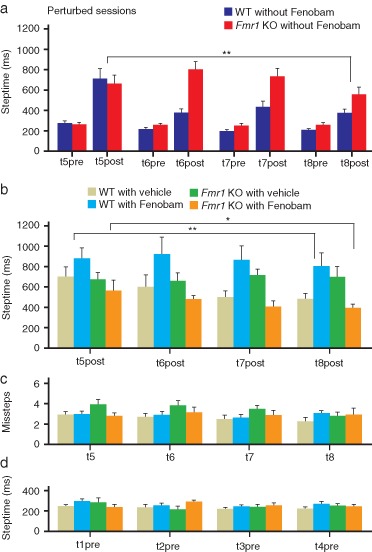
Deficits of procedural memory formation in *Fmr1* KO mice and rescue by Fenobam (a) Motor learning was tested with the use of the Erasmus Ladder by calculating for every associative motor learning session (‘perturbed sessions'; (t5post to t8post) the average duration of the step that immediately followed the onset of the US (‘post-steptime’). As control, we also calculated the average duration of the step that immediately preceded the onset of the US (‘pre-steptime’). *Fmr1* KO (*n* = 16) mice showed a specific deficit in procedural memory formation in that they showed longer post-steptimes to an auditory conditioned stimulus than their WT littermates (*n* = 17) (*P* = 0.002; repeated measures anova). The differences in post-steptimes between *Fmr1* WT and KO mice did not depend on differences in the pre-steptime values (*P* = 0.085; repeated measures anova). (b) *Fmr1* KO mice injected with Fenobam (*n* = 10) showed faster post-steptime responses than *Fmr1* KO mice that receive vehicle (*n* = 9) (*P* = 0.046; LSD *post hoc* test). Moreover, they were indistinguishable from vehicle-treated WT mice (*n* = 11) (*P* = 0.303; LSD *post hoc* test). In contrast, Fenobam had a negative side effect on WT mice (*n* = 13) in that it increased their post-steptimes compared with the animals that were injected with vehicle (*P* = 0.004; LSD *post hoc* test). (c), (d) Interestingly, the number of missteps and pre-steptime responses during the same period of training were not affected by the administration of Fenobam neither in *Fmr1* KO nor in WT mice (missteps *P* = 0.234; pre-steptime *P* = 0.463; repeated measures anova). Error bars represent standard error of the mean. Asterisks indicate level of significance: * stands for *P* < 0.05; ** stands for *P* < 0.01; *** stands for *P* < 0.001.

**Figure 3:**
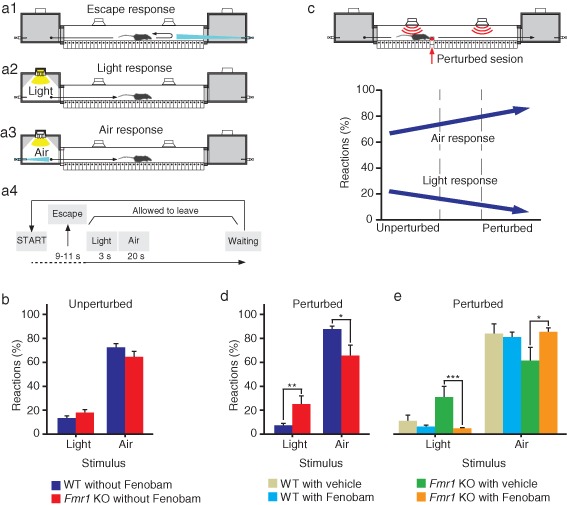
Avoidance discrimination impairments of *Fmr1* KO mice measured with the Erasmus Ladder Light and air stimuli are used to control the moment of departure. (a1) When the mouse leaves the starting box before the light turns on, the crosswind is turned on at full force (30 km/h) from the opposite box. This wind usually causes the mouse to immediately return to the starting shelter. (a2) When the shelter light is turned on the mouse is allowed to walk on the ladder. The light will remain on until the mouse reaches the end box. Permitted ladder crossing is accompanied by a tailwind that is kept constant at 16 km/h at the actual position of the mouse. (a3) If the mouse does not leave the starting box within 3 seconds after the light is turned on, an air puff comes from the pressurized air outlets in this shelter. Normally, the air puff encourages the mouse to leave the starting box. (a4) A schematic representation of the temporal order of events is mentioned under a1–a3. (b) During unperturbed sessions there was no difference in the percentage of reactions, neither to the light nor to the air stimuli, between *Fmr1* KO mice (*n* = 32) and their WT littermates (*n* = 41) (light: *P* = 0.136; air: *P* = 0.154; one-way anova). (c) The percentage of times that WT mice properly responded to the light stimulus decreased when they were transferred from the unperturbed to the perturbed sessions. The opposite occurred with the percentage of reactions to the air stimulus. (d) *Fmr1* KO mice (*n* = 13) reacted significantly more to the light and less to the air stimuli than WT mice (*n* = 17) (light: *P* = 0.006; air: *P* = 0.007; one-way anova). Fenobam-treated *Fmr1* KO mice (*n* = 10) decreased their response to the light and increased their response to the air stimuli with respect to vehicle-treated *Fmr1* KO mice (*n* = 9) (light: *P* = 0.001; air: *P* = 0.013, LSD *post hoc* tests); furthermore, their responses to light and air stimuli were similar to that of the vehicle-treated WT mice (*n* = 11) (light: *P* = 0.309; air: *P* = 0.975, LSD *post hoc* tests). Error bars represent standard error of the mean. Asterisks indicate level of significance: * stands for *P* < 0.05; ** stands for *P* < 0.01; *** stands for *P* < 0.001.

Apart from motor coordination deficits, the Erasmus Ladder is able to detect cognitive phenotypes in mice. Cognition is tested by determining the capability of mice to respond to the cues of departure (light or air) and to modify this response under certain circumstances. Thereby, unperturbed sessions that are neutral in the beginning of the experiment (Fig. 1), turn into unpleasant, perturbed sessions (Figs 2 and 3), which will reverse the initial response of mice to the given cues. Indeed, in the attempt to reduce their exposure to the US stimulus during perturbed sessions, mice inhibit the reaction to the cues of departure (‘avoidance behavior’). At the beginning of each session a mouse was placed in the starting box and after a period (randomly varying from 9 to 11 seconds) the shelter light is turned on (first cue). At this stage the mouse is supposed to leave the box. In case the mouse left the box before the light turned on (so-called ‘escape behavior’), a strong air puff from the opposite box drives the mouse back into the shelter, and a new cycle begins. If the mouse does not leave the box within 3 seconds after the onset of the light, a strong air puff is given from the pressurized air outlets in the box (second cue) so as to push the mouse out of the box within 20 seconds. When the mouse arrives at the opposite shelter, the pressurized air outlets and light are switched off, and after a period randomly varying from 9 to 11 seconds, the cycle is repeated. Thus, each trial of the training paradigm described above can result in one out of four possible outcomes: (1) the mouse leaves the box before the light is turned on (‘escape response’) (Fig. 3a1); (2) the mouse responds to the light and leaves the box on time (‘light response’) (Fig. 3a2); (3) the mouse does not respond to the light, but responds to the strong air pressure and leaves the box on time (‘air response’) (Fig. 3a3) and (4) the mouse neither responds to the light nor to the air within the allotted time period and has to wait for another cycle (‘waiting response’). A schematic description of the possible outcomes and their interactions on time is depicted in Fig. 3a4. During each session, we quantified the percentage of light responses, air responses and escape, and used them to assess the mouse's cue response capabilities and avoidance behavior.

### Statistical analysis

Off-line analyses of motor coordination and avoidance behavior were performed using custom written software in Labview (National Instruments) and the results were stored in a relational database (MySQL). Statistical tests were performed with SPSS Statistics (IBM Corporation, New York, NY, USA). Data were compared using one-way analysis of variance (anova) or two-way repeated measures anova, as appropriate. If a significant difference was found, *post hoc* analysis was performed using Fisher's least significant difference (LSD) test, unless stated otherwise. In total, 39 *Fmr1* KO and their 41 WT littermates were measured in the Erasmus Ladder. Four mice were excluded from the analysis of motor performance and motor learning because the computer failed capturing several post-steptime values that could have changed the averaged post-steptime values. Three mice were excluded from the avoidance behavior analysis because they were not measured properly.

## Results

### *Fmr1* KO mice show no deficits in motor performance

To rule out the potential caveat that possible differences in motor learning or avoidance discrimination are partly due to differences in motor performance, we started out by testing the overall motor performance level on the Erasmus Ladder. Unperturbed sessions (s1–s4), that were used to evaluate motor performance capabilities, showed that *Fmr1* KO mice (*n* = 35) and their WT littermates (*n* = 41) did not show significant differences in steptimes (genotype: *F*_1,74_ = 0.06, *P* = 0.810; repeated measures anova) (Fig. 1). In addition, the number of missteps in *Fmr1* KO mice was not significantly different from that in WTs (genotype: *F*_1,74_ = 1.65, *P* = 0.204; repeated measures anova) (data not shown).

### *Fmr1* KO mice show deficits in procedural memory formation, which can be rescued by Fenobam

In contrast to WT mice (*n* = 17), which learned to adjust their walking pattern to the CS over the sessions, *Fmr1* KO (*n* = 16) mice did not learn to do so (genotype × session: *F*_3,93_ = 5.33, *P* = 0.002; repeated measures anova) (Fig. 2a). Already during the second perturbed session, WT mice showed decreased post-steptimes (t6post: 374.24 ± 37 ms vs. t5post: 711.12 ± 99 ms, *P* < 0.001; one-way anova with Dunnett's *post hoc* test). The *Fmr1* KO mice did not show any motor learning during the perturbed sessions (*F*_3,60_ = 0.16, *P* = 0.160; one-way anova) (Fig. 2a). Even during the fourth perturbed session, *Fmr1* KO mice did not show a clear reduction in post-steptimes compared with the first perturbed session (t8post: 557.97 ± 72 ms vs. t5post: 663.84 ± 82 ms). The difference in post-steptimes between WT and *Fmr1* KO mice did not depend on a difference in steptimes before the onset of the CS–US stimuli as shown by the pre-steptime values which were not statistically different (genotype × session: *F*_3,93_ = 2.27, *P* = 0.085; repeated measures anova) (Fig. 2a).

To explore the effect of an mGluR5 inhibitor on procedural memory formation we administrated (30 min before each perturbed session) Fenobam or MC as vehicle to both *Fmr1* KO and WT mice. The interaction genotype × treatment was statistically different (genotype × treatment: *F*_1,39_ = 9.29, *P* = 0.004; repeated measures anova). *Fmr1* KO mice treated with Fenobam (*n* = 10) showed faster post-steptime responses during the perturbed sessions than those that were injected with vehicle (*n* = 9) (*P* = 0.046; LSD *post hoc* test) (Fig. 2b). Moreover, they were indistinguishable from WT mice treated with vehicle (*n* = 11) (*P* = 0.303; LSD *post hoc* test) (Fig. 2b). This improvement was not due to the injection itself, because vehicle-treated *Fmr1* KO were not significantly different from those that did not received treatment (*P* = 0.955; LSD *post hoc* test). The overall level of motor performance remained intact during the perturbed sessions in that there was no significant interaction genotype × treatment neither in the number of (genotype × treatment: *F*_1,39_ = 1.46, *P* = 0.234; repeated measures anova) (Fig. 2c) nor in pre-steptime responses (genotype × treatment: *F*_1,39_ = 0.549, *P* = 0.463; repeated measures anova) (Fig. 2d).

### Fenobam elicits negative side effects in wild types

In contrast, the motor learning in Fenobam-treated WT (*n* = 13) was severely impaired. In this group, post-steptimes increased significantly compared with animals that were not treated (*P* < 0.001; LSD *post hoc* test) or that received vehicle (*P* = 0.004; LSD *post hoc* test) (Fig. 2b). Again, this was not due to the injection, because vehicle-treated WT mice showed normal post-steptimes compared with nontreated animals (*P* = 0.305; LSD *post hoc* test) (data not shown).

### *Fmr1* KO mice show abnormal avoidance behavior, which can be rescued by Fenobam

During unperturbed sessions WT (*n* = 41) and *Fmr1* KO (*n* = 32) mice used light and air as cues to leave the box and to start to walk on the ladder at a similar level (light genotype: *F*_1,71_ = 2.27, *P* = 0.136; air genotype: *F*_1,71_ = 2.07, *P* = 0.154; one-way anova) (Fig. 3b). However, during perturbed sessions, WT mice reacted significantly less to light and significantly more to air stimuli than during the unperturbed sessions (light phase: *F*_1,56_ = 5.86, *P* = 0.019; air phase: *F*_1,56_ = 11.79, *P* = 0.001; one-way anova). These changes reflect avoidance behavior in order to delay the exposure to the perturbation. In *Fmr1* KO mice this avoidance behavior did not occur; they did not show any change in their reactions, neither to the light nor to the air stimuli (light phase: *F*_1,43_ = 0.99, *P* = 0.326; air phase: *F*_1,43_ = 0.009, *P* = 0.763; one-way anova) (Fig. 3b,d); in line with these results, they also reacted more to the light and less to the air stimuli than their WT littermates during the perturbed sessions (light genotype: *F*_1,28_ = 8.67, *P* = 0.006; air genotype: *F*_1,28_ = 8.41, *P* = 0.007; one-way anova) (Fig. 3d). Importantly, the administration of Fenobam rescued the abnormal avoidance behavior of *Fmr1* KO mice. The interaction genotype × treatment was significant for both light and air stimuli (light genotype × treatment: *F*_1,39_ = 4.85, *P* = 0.034; air genotype × treatment: *F*_1,39_ = 4.36, *P* = 0.043; one-way anova). *Fmr1* KO mice treated with Fenobam decreased the percentage of occasions that they reacted to the light stimulus with respect to vehicle-treated *Fmr1* KO mice from 29.78% ± 8.72 to 4.46% ± 0.73 (*P* = 0.001; LSD *post hoc* test) and significantly increased their percentage of responses to the air stimulus from 57.77% ± 10.11 to 82.35% ± 4.17 (*P* = 0.013; LSD *post hoc* test) during perturbed sessions; moreover, they were not significantly different from vehicle-treated WT mice (light: *P* = 0.309; air: *P* = 0.975; LSD *post hoc* test) (Fig. 3e). This improvement was due to the administration of Fenobam and not due to the injection itself, because vehicle-treated *Fmr1* KO mice showed the same response to the light and air as *Fmr1* KO mice that did not receive any treatment (light: *P* = 0.407; air: *P* = 0.540; LSD *post hoc* tests).

## Discussion

Subjecting *Fmr1* KO mice to treatment with Fenobam while performing locomotion conditioning tasks showed that the Erasmus Ladder can be used to test different types of learning simultaneously and to assess the impact of different drugs at a medium to high throughput level. Our main findings are that *Fmr1* KO mice show deficits in both associative motor learning and avoidance behavior and that Fenobam, which is a selective mGluR5 inhibitor, can rescue both deficits. In addition, we show that Fenobam treatment of WT mice showed profound side effects in the motor coordination task. Together, these findings are in line with the mGluR hypothesis, and they offer, as will be discussed below, impetus to potential therapies of both motor and cognitive symptoms in FXS patients.

*Fmr1* KO mice showed a marked deficit in procedural memory formation in that they did not reduce significantly their post-steptime response during the perturbation training sessions. Given that there were no differences in steptimes during the unperturbed sessions, we can conclude that the higher values of post-steptimes of *Fmr1* KO mice were not due to motor performance deficits. The deficits in locomotion conditioning on the Erasmus Ladder agree with the findings by Koekkoek *et al.* (2005) in which it was shown that *Fmr1* KO mice and FXS patients show deficits in classical delay eyeblink conditioning. The abnormalities of the *Fmr1* KO mice in locomotion conditioning were rescued after administration of 30 mg/kg of Fenobam, 30 min before the learning task started. In fact, *Fmr1* KO mice were able to reduce significantly their post-steptime response to an auditory CS, achieving values that were close to those of WT mice. As application of vehicle (MC alone) did not significantly affect motor behavior in the *Fmr1* KO mice, it is parsimonious to conclude that the therapeutic impact of Fenobam in *Fmr1* KO mice on procedural memory formation was not due to the induction of stress or other sham effects that might have been induced by the injection itself.

*Fmr1* KO mice also showed a marked deficit in avoidance behavior in that they did not show the normal waiting reaction inside the box; they did not delay the exposure to the suddenly occurring aversive stimulus, i.e. the perturbation due to the rising rung. WT mice tend to remain inside the boxes as long as possible during the unpleasant perturbation sessions. They usually do not leave the box when the light is turned on and they need the air stimulus significantly more often to force them out of the box as compared to the unperturbed performance task. *Fmr1* KO mice instead did not modify their responses to the light and air departure cues after they were transferred from the unperturbed to the perturbed sessions. It appeared as if they did not fully perceive and/or did not know how to react to a stressful situation. This type of apathetic reaction might point toward a decrease in anxiety and/or a decrease in fear memory. This possibility is in line with other FXS mouse model studies in which a general lower level of anxiety and/or general deficits in fear memory formation were observed (Liu & Smith 2009). Importantly, administration of Fenobam also rescued this cognitive phenotype. These results agree with the reports that showed positive effects of mGluR5 inhibitors on abnormalities in prepulse inhibition (PPI) in FXS patients (Berry-Kravis *et al.* 2009) and *Fmr1* KO mice (de Vrij *et al.* 2008).

While *Fmr1* KO mice improved both their procedural memory formation and avoidance discrimination after administration of Fenobam, WT mice showed a clear negative side effect in that their procedural memory formation was severely impaired. Similarly, Jacob *et al.* reported impairments in the passive avoidance test, the Morris water maze and contextual fear conditioning following administration of similar dosages of Fenobam (Jacob *et al.* 2009). These findings emphasize the critical status of a proper diagnosis for patients with intellectual disability. While patients with FXS may benefit from mGluR5 inhibitors without overt negative side effects (Berry-Kravis *et al.* 2009), other patients with different forms of intellectual disability may suffer profoundly from inadequate treatment with drugs like Fenobam.

## Conclusions

Testing locomotion conditioning and avoidance discrimination in mutant mice can be performed reliably and noninvasively with the use of the Erasmus Ladder, and the impact of drugs on these tests can be screened at a medium to high throughput level. *Fmr1* KO mice show deficits in both procedural memory formation and avoidance discrimination and both deficits can be rescued with Fenobam. On the basis of this mouse model study, it can be inferred that the use of mGluR inhibitors may be beneficial for procedural memory formation and avoidance discrimination in FXS patients, but it appears contraindicated for healthy controls or non-FXS patients with intellectual disability.
